# A Widespread and Unusual RNA Trans-Splicing Type in Dinoflagellate Mitochondria

**DOI:** 10.1371/journal.pone.0056777

**Published:** 2013-02-20

**Authors:** Christopher J. Jackson, Ross F. Waller

**Affiliations:** School of Botany, The University of Melbourne, Victoria, Australia; University of Surrey, United Kingdom

## Abstract

Cytochrome oxidase subunit 3 (Cox3) is a mitochondrion-encoded core membrane protein of complex IV of the mitochondrial respiratory chain, and consists of seven trans-membrane helices. Here we show that in diverse later-branching dinoflagellates, *cox3* is consistently split into two exons in the mitochondrial genome between helices six and seven. Gene exons are transcribed as two discrete oligoadenylated precursor RNAs, and these are subsequently trans-spliced to form a complete coding mRNA. This trans-splicing is highly unusual in that some of the oligoadenylated tail is incorporated at the splice site, such that a short string of adenosines links the two coding exons. This feature is consistently represented in diverse dinoflagellates, however the number of adenosines added varies according to the size of the coding gap between the two exons. Thus we observed between zero (*Amphidinium carterae*) and 10 (*Symbiodinium* sp.) adenosines added in different taxa, but the final coding sequence length is identical with the reading frame maintained. Northern analyses show that precursor *cox3* transcripts are approximately equally abundant as mature *cox3* mRNAs, suggesting a slow or regulated maturation process. These data indicate that the splicing mechanism in dinoflagellate mitochondria is tolerant of variations in the length of the precursor coding sequence, and implicates the use of a splicing template, or guide molecule, during splicing that controls mature mRNA length.

## Introduction

The expression pathway from gene to protein is not always a simple one. One of the most common elaborations on the gene→transcript→protein dogma is the presence of introns which break up otherwise contiguous coding sequences within a genome, and which must be removed by cis-splicing of the gene transcript [1]. A rarer form of gene interruption is when gene exons are more distantly separated on the genome and/or encoded on opposite strands, dictating that individual exons are separately transcribed. In these cases, re-constitution of complete coding mRNAs requires a process of trans-splicing of the transcript exons.

In organelles (plastids and mitochondria), the most prevalent form of RNA trans-splicing known occurs via discontinuous group I and II introns. These two intron families differ in the chemistry of their splicing reactions, but in both cases splicing involves the formation of a catalytic secondary structure by the intron sequence itself [2], [3]. Thus, for discontinuous introns inter-molecular base pairing of the partial group I or II intron sequences regenerates the required catalytic function, enabling the trans-splicing of exons. Most known organelle trans-splicing examples involve group II introns in the chloroplasts and mitochondria of plants and green algae, but discontinuous group I introns have also been reported from plants and early-branching animals (placazoans) [4]–[6]. Nucleus-encoded genes can also undergo trans-splicing, and two broad splicing categories can be defined: the joining of separate protein-coding exons via fragmented spliceosomal introns, and the splicing of a short UTR exon onto the 5′ end of gene transcripts (spliced-leader (SL) trans-splicing) [1]. The former type is found rarely, with examples from *Drosophila* [7] and the protist *Giardia intestinalis* [8], [9], whereas SL splicing is found broadly in eukaryotes including many metazoans, as well as protists such as dinoflagellates, diplonemids, and kinetoplastids [10]–[12]. Both nuclear trans-splicing types rely on elements of the same spliceosomal machinery involved in classical intron removal via cis-splicing [12]. In dinoflagellates, SL trans-splicing occurs throughout dinoflagellate diversity, including the basal species *Hematodinium* sp., *Oxyrrhis marina* and *Perkinsus marinus* [13]–[15]. The dinoflagellate SL transcript is ∼50–60 nucleotides long, and contains a 22-nucleotide exon at the 5′ end as well as downstream intron sequence. A conserved spliceosomal binding site occurs in the exon sequence, and the trans-splicing reaction apparently utilizes canonical GU-AG intron boundaries, with the GU donor dinucleotide encoded on the SL transcript intron. Dinoflagellate SL splicing is thought to be catalysed by components of the nuclear spliceosome [11]. Yet another type of trans-splicing occurs in the tRNA genes of Archaea, and involves reconstitution of introns characterised by a bulge-helix-bulge (B-H-B) motif at the intron-exon junctions; unlike the cases above, removal of B-H-B introns requires an endonuclease and a ligase [16].

Recently a further example of RNA trans-splicing has emerged, occurring in the mitochondrion of the dinoflagellate *Karlodinium veneficum* (synonym: *K. micrum*) [17], [18]. While dinoflagellate mitochondrial genomes are among the smallest known in terms of gene content, encoding a paltry three proteins, these genomes are otherwise highly complex. The genes occur in multiple copies including numerous and variously fragmented forms, suggesting a genome that is highly recombinatorial [18], [19]. For one of the *K. veneficum* mitochondrial genes, *cox3*, no intact gene remains on this genome. Despite this, complete transcripts of *cox3* have been detected as oligoadenylated cDNAs, implying that the *cox3* gene exons are transcribed and trans-spliced together to generate a complete mRNA [17]. Consistent with this, transcriptome data additionally reveal an oligoadenylated but truncated transcript encoding the first 85% (nucleotides 1–731) of this gene, corresponding to the largest *cox3* gene fragment found in the genome. The remainder of *cox3* occurs as a separate gene fragment (nucleotides 737–858), and a transcript of this fragment was presumed to complete the mRNA [17], [18]. Two features of this trans-splicing case are unusual: 1) no genomic sequence around the splice sites could be identified that could participate in a known splicing reaction such as group I/II intron fragments, or bulge-helix-bulge formation; and 2) five, non-encoded adenosine nucleotides bridge the gap in *cox3* transcripts between the two gene exons (nts 1–731, 737–858), presumably donated from the oligoadenosine tail of the 731-nucleotide transcript [17]. In this report we describe an unusual partial conservation of this splicing reaction seen across diverse dinoflagellates that provides insight into the novelty of this splicing mechanism.

## Methods

### Cell Culture, Nucleic acid Extraction, cRT-PCR

*Karlodinium veneficum* (strain CCMP415), *Alexandrium catenella* (strain ACPP01), *Amphidinium carterae* (strain CCMP121) and *Symbiodinium* sp. (strain Tc 13) were cultured in Guilard’s f2 media at 16°C (*K*. *veneficum* and *A*. *carterae*) or 25°C (*Symbiodinium* sp. and *A*. *catenella*) on a 12-h light/12-h dark cycle. Cells were harvested by centrifugation (10 min, 2,600 g), and total RNA was extracted using Trizol (Invitrogen). For each species, ∼750 ng of otherwise untreated total RNA was treated with DNase I (Invitrogen). Subsequently, each RNA sample was ligated head to tail using an RNA ligase (Promega), according to the manufacturer’s instructions in a total volume of 40 µL (∼10 µL DNAse-treated RNA sample, 20 µL PEG 8000, 4 µL T4 RNA ligase buffer, 1 µL RNasin® Ribonuclease Inhibitor, 1 µL/10 units T4 RNA ligase, 4 µL nuclease-free water, incubated at 37°C for 30 mins). First strand cDNA synthesis across the ligated mRNA ends was performed for *cox3* using SuperScript III reverse transcriptase (Invitrogen) according to the manufacturer’s instructions, using 10 µL of ligated RNA as template for each 20 µL reaction (primer for *K*. *veneficum cox3H7*: KVcox3H7rev (AACTCTTAAATTTAAAAACCAAAC); *Symbiodinium* sp. and *A*. *catenella cox3H7*: SspAcatcox3H7rev (GATTATAAAATAAATGAACTTCTGA); *A*. *carterae cox3H7*: Acarcox3H7rev (CAAGCAAAAAATAAATGTACTTCTG); *K. veneficum*, *Symbiodinium* sp. *cox3H1-6*: KVcox3H1-6rev (AGACAAAATGCACCTGATGC); *A. catenella cox3H1-6*: Acatcox3H1-6rev (AATCTGATGCAACTTCCAGATG); *A. carterae cox3H1-6*: Acarcox3H1-6rev (GCAAAATACATAGAATAAAACAGG). Subsequently, PCR was performed with Phusion ® High-Fidelity DNA polymerase (NEB) (2 µL cDNA template, initial denaturation 98°C 2 mins, then 35 cycles of 98°C 30 secs, 55°C 30 secs, 72°C 1 min ) using primers directed outward toward the gene termini (*K. veneficum cox3H7*: KVcox3H7rev and KVcox3H7for (AATCTTATGGTTATTTATCTTTC); *Symbiodinium* sp. and *A*. *catenella cox3H7*: SspAcatcox3H7rev and SspAcatcox3H7for (AATTTCTATTGGCATTTTCTTG) or Kvcox3H7for (for *A*. *catenella* only); *K. veneficum*, *Symbiodinium* sp. *cox3H1-6*: KVcox3H1-6rev and KVcox3H1-6for (TTTCTTTCATCTTGTCGTTGG); *A. catenella coxH1-6*: Acatcox3H1-6rev and KVcox3H1-6for; *A. carterae cox3H1-6*: Acarcox3H1-6rev and Acarcox3H1-6for (TTTCTTTCACCTTATTGTTGG); *A*. *carterae cox3H7*: Acarcox3H7rev and Acarcox3H1-6for (TTTATTGGCATTTTGTTGAGG). As primers to *cox3* precursors also bound to full-length *cox3* transcripts, gels of cRT-PCR products contained larger bands corresponding to head-to-tail ligated full-length *cox3* molecules, with sequence spanning the splice site. For *A. catenella* and *A. carterae* these larger bands were cloned, whereas cDNAs for *K*. *veneficum cox3* (strain CCMP415) were available from a previously constructed cDNA library [20]. PCR products were ligated into the pGEM T-easy vector (Promega), cloned, and fully sequenced.

### Northern Blot Analysis

Hybridization probe templates for *K*. *veneficum cox3H1-6* and *cox3H7* were generated using PCR from a full-length cDNA cloned into pGEM-T Easy vector (*cox3H1-6* primers: KvH1-6ProbeF (AGTATTCATCAGGAAGTTGC) and KvH1-6ProbeR (TTAGAAGAAGAAGACCAACGAC); *cox3H7* primers: KvH7ProbeF (TTGGTTTTTAAATTTAAGAG) and KvH7ProbeR (ATAACGAGTAAAGGAATAGAAAG). PCR fragments were purified from gels and random hexamer-based probes were constructed using the Prime-a-gene labeling system (Promega) and ^32^P-labeled dATP, according to the manufacturer’s instructions. Total RNA (5µg per lane) was separated on a 4% polyacrylamide/urea gel (per 5 mL of gel solution: 0.5 mL 10X Tris/Borate/EDTA buffer, 3.5 mL 10M urea, 0.5 mL 40% 19∶1 Acrylamide/Bis solution, 50 µL 10% ammonium persulphate, 450 µL water, 5 µL TEMED) at 150V in 1X TBE running buffer (Mini-Protean® 3 Cell, Biorad). Separated RNA was transferred to Hybond N+ membrane (GE Healthcare) via electroblotting with 0.5X TBE transfer buffer, at 80 volts for 1 hour at 4°C, (Mini Trans-Blot® Electrophoretic Transfer Cell, Biorad), and RNA was cross-linked by UV irradiation. Membrane blocking was performed with modified Churches buffer (51 g Na2HPO4.2H20, 16.8 g anhydrous NaH2PO4, 4 ml of 0.5 M ethylenediaminetetraacetic acid, and 70 g SDS per liter) for 2 hours at 65°C. Probe hybridization was performed overnight at 65°C in modified Churches buffer. Following hybridization, membranes were washed twice for 5 min each in 4×SSC +0.5% SDS then with the following series: 2×SCC +0.5% SDS; 4×SSC +0.5% SDS; 2×SCC +0.5% SDS; 4×SSC +0.5% SDS. All wash steps were carried out for 1 h at 65°C. Membranes were visualized using X-ray film, (exposure time ∼1 hour).

## Results and Discussion

**Figure 1:**
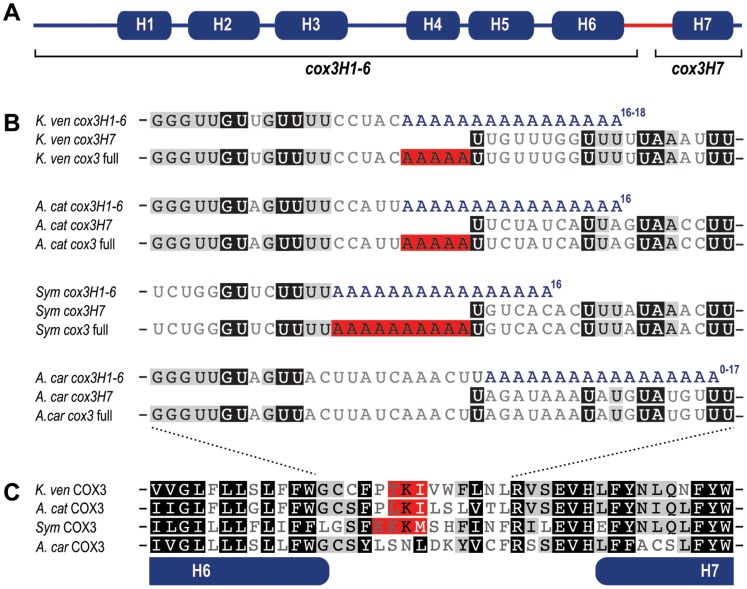
*cox3* trans-splicing in diverse dinoflagellates. *A.* Schematic of dinoflagellate Cox3 showing seven predicted trans-membrane helices encoded by fragmented *cox3* coding sequences *cox3H1-6* and *cox3H7*. *B.* Alignment of nucleotide sequence at the splice site of transcript precursors *cox3H1-6* and *cox3H7*, and the splice product *cox3*. Corresponding sequences are shown for *Karlodinium veneficum* (*K. ven*), *Alexandrium catenella* (*A. cat*), *Symbiodinium* sp. (*Sym*) and *Amphidinium carterae* (*A. car*). The range of lengths observed for oligoadenylated tails on *cox3H1-6* is shown in superscript. Red highlighting indicates A nucleotides from the oligoadenylated tail incorporated into the *cox3* splice product. *C.* Dinoflagellate Cox3 amino acid sequence alignment at the splice site between helices 6 and 7. Amino acid codons determined by inclusion of oligoadenylation nucleotides are shown with red highlighting.

The *cox3* gene codes for cytochrome oxidase subunit 3 (Cox3) of complex IV of the mitochondrial electron transport chain. The majority of this membrane protein is made up of seven trans-membrane spanning helices (Fig. 1A) [21]. The break in coding sequence in *K. veneficum cox3* occurs between transmembrane helices six and seven, so we define the two gene exons as *cox3H1-6* (helix 1 to 6), and *cox3H7* (helix 7). To unambiguously characterise the length and sequence of precursor transcripts from these two genes, and the resultant full-length *cox3* transcript, we used circular reverse transcription PCR (cRT-PCR) [22]. This technique uses RNA ligase to circularise RNA molecules harvested from cells, and then outward-orientated primers are used to RT-PCR amplify and sequence the joined ends. The presence of 3′ oligoadenylation enables the 3′-terminus of the transcript to be identified where it joins the 5′-terminus. Multiple, independent cRT-PCR generation of *cox3H1-6, cox3H7,* and *cox3* transcripts confirmed that this technique faithfully identifies the mRNA ends (Data S1).

These cRT-PCR data revealed that precursor transcripts *cox3H1-6* and *cox3H7* correspond precisely to the respective sequence components of the complete *cox3* transcript. The 5′ end of *cox3H1-6* is exactly the same length as *cox3*, and the 5′ end of *cox3H7* ends at the nucleotide 737, the exact position where it is subsequently joined to the *cox3H1-6* transcript (Fig. 1B). The 3′ end of *cox3H1-6* is oligoadenylated at position 731 (as previously described; Fig. 1B), and cRT-PCR shows that it receives between 16–18 A nucleotides. The 3′ end of *cox3H7* matches the full-length *cox3* end precisely in sequence and oligoadenylation site, and both bear 13–16 A nucleotides. These data suggest that the dominant precursor species contain only sequence that will be incorporated into the complete *cox3* mRNA.

To explore the novelty of this trans-splicing process seen in *K. veneficum*, we have examined transcripts of *cox3* in three further dinoflagellate taxa - *Alexandrium catenella*, *Symbiodinium* sp., and *Amphidinium carterae* - that represent a broad range of dinoflagellate diversity. cRT-PCR was used to recover transcripts of *cox3* sequence and to characterise their lengths and transcript termini (Fig. 1B). Similar to *K. veneficum*, all new taxa show evidence of trans-splicing by the presence of truncated transcripts equivalent to *cox3H1-6* and *cox3H7*, as well as a full-length *cox3*. The 5′ end of *cox3H7* is conserved in length in all four taxa, despite sequence variation in the first eight nucleotides (Fig. 1B, Data S1). In all cases splicing occurs directly onto the first nucleotide of this transcript, which is a U in every case. The 3′ boundary of *cox3H1-6*, however, is variable. While *A. catenella cox3H1-6* is oligoadenylated at precisely the same position as *K. veneficum*, *Symbiodinium* sp. is oligoadenylated at a position five nucleotides earlier, and *A. carterae* six nucleotides later (Fig. 1B). This variation, however, does not affect the mature *cox3* length. The five nucleotide coding gap in *A. catenella* is filled with five A nucleotides exactly as for *K. veneficum*, presumably from the oligoadenosine tail. In *Symbiodinium* sp. the gap of 10 nucleotides is filled with 10 A nucleotides. In *A. carterae*, where no coding gap exists, splicing occurs one nucleotide upstream of the oligoadenosine tail so no non-coded A nucleotides are included (Fig. 1B). The length of oligoadenylation observed for all taxa and all *cox3* products is similar, typically ranging from 12–19 nucleotides. For *cox3H1-6* this is sufficient to span the respective coding gaps between exons.

The sequence termini of *cox3* precursor transcripts and positions of oligoadenylation seen in the cRT-PCR data are corroborated by available transcriptome data. For example, the *cox3H1-6* oligoadenylation sites (Fig. 1B) are identical in *K. veneficum* EST sequences [17], and from *Symbiodinium* sp. eight ESTs precisely match the *cox3H7* 5′ sequence (accessions; FE537727, FE537728, FE537811, FE537812, FE537869, FE537870, FE538147, FE538148). We did, however, recover some cRT-PCR data that showed some termini variation (Data S1). In *K*. *veneficum cox3H7*, two of six independent cRT-PCR products bore an additional 15 nucleotides at the 5′ terminus **(**UUCCAAGAAAAGCCU**)**. This extra tag lacks any complementarity with *cox3* coding sequence, BLAST searches did not recover matches to *K*. *veneficum* mitochondrial genomic sequence [17], and RT-PCR could not reproduce a *cox3H7* fragment linked to this extension. Similarly, in *Symbiodinium* sp., one of seven *cox3H7* amplicons is 5′ truncated by 10 nucleotides, relative to the other six sequences. These data are consistent with previous evidence of dinoflagellate mitochondrial transcripts occasionally occurring either fused to unrelated sequence, or truncated [23], and likely represent non-functional transcript species. In *A*. *carterae*, of three *cox3H1-6* cRT-PCR amplicons, one lacks an oligo-A tail, and another is oligoadenylated one nucleotide earlier (c.f. Fig. 1B). Neither of these two variations would directly affect the sequence of complete *cox3* as they occur downstream of the splice site, and therefore such variation in *A. carterae* might be tolerated.

Post-transcriptional RNA end capping has been described in some dinoflagellate organelles, but we observe no evidence of such modification to any of the *cox3* transcripts. In the deep-branching dinoflagellate *Oxyrrhis marinus* 5′ capping by addition of 8–9 U nucleotides to mitochondrial protein-encoding transcripts has been reported, and in dinoflagellate plastids mRNAs gain 3′ polyuridine tracts of up to 40 nucleotides after transcription [24]–[26]. Both of these additions are detectible by cRT-PCR [24], [26], but were not observed in *cox3* transcripts for any of the taxa examined. Further capping reactions that modify the 5′-phosphate group on RNA molecules, such as the modified guanine nucleotide added to the 5′ end of most eukaryotic nuclear transcripts [27], would prevent RNA ligation and detection by cRT-PCR. While such capping is not known from either bacteria or mitochondria, it remains possible that further *cox3* transcript species might exist in addition to those detected by cRT-PCR and transcriptomics approaches.

**Figure 2:**
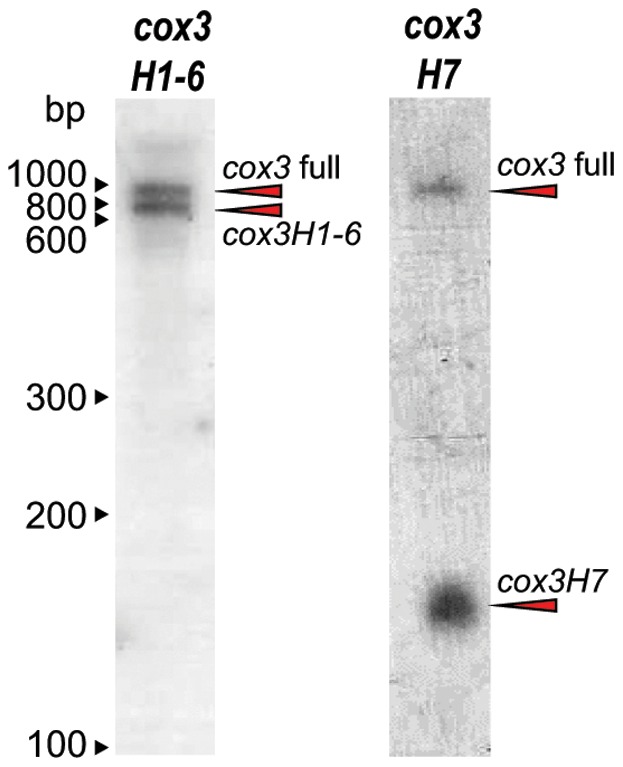
Northern blot analysis of *K. veneficum cox3H1-6*, *cox3H7* and full-length *cox3* transcripts. Total *K. veneficum* RNA was hybridized with either a probe corresponding to the *cox3H1-6* or *cox3H7* sequence. Bands observed correspond in size to the precursor molecules *cox3H1-6* (∼745 nt) and *cox3H7* (∼136 nt), along with full length *cox3* (∼872 nt) (note: predicted RNA lengths include oligoadenylation tails).

To examine the relative abundance of *cox3H1-6* and *cox3H7* transcripts in comparison to full-length *cox3* in dinoflagellate mitochondria, we performed Northern blot analysis of *K. veneficum* total RNA. Probes were made corresponding to either *cox3H1-6* or *cox3H7*. Each would therefore detect the respective precursor and also the full-length *cox3* transcript, enabling relative steady-state quantitation of these species before and after splicing. Indeed, two bands were detected in Northern blots for each probe, and in each case these bands corresponded in size to the respective precursor and full-length *cox3* (Fig. 2, arrowheads). The two bands detected by *cox3H1-6* are of approximately equal abundance, whereas the *cox3H7* precursor band is even more abundant than the full-length band detected by this probe. Together, these Northern blots indicate that rather than precursor transcripts being very minor components of the total RNA pool, they are present in similar amounts to full length *cox3* mRNA. The high relative abundance of precursors suggests either a slow rate of trans-splicing, or a regulated process that maintains a large pool of precursors. We tested to see if compounds that are known to perturb mitochondrial electron transport (antimycin A and Salicylhydroxamic Acid (SHAM) [28]), would lead to changes in the relative abundances of *cox3* precursors, but found no evidence of such regulation in these experiments (not shown). A further result of the Northern blots was lack of evidence of additional *cox3* size species as prevalent transcripts. Polycistronic transcript sequence has previously been detected in dinoflagellate mitochondria [17], [23], [29], [30], and generation of large transcripts from few promoters is quite common in mtDNAs where large precursor RNA molecules are processed to generate individual gene transcripts [31]. If *cox3* precursor transcripts are similarly generated by processing large polycistronic transcripts, then processing to the final precise lengths must be fast enough that little intermediate is evident by Northern blot detection or sequencing methods described above. Alternatively, it is possible that the *cox3* precursors could be transcribed as their final lengths; we presently have no data that can discern between these scenarios.

A consequence of abundant precursor transcripts is that these would need to be excluded from the downstream expression machinery, namely translation. However, we detected no obvious differentiation of precursor versus complete transcript, such as post-transcriptional modifications or oligoadenylated tail length differences, that might distinguish precursors from mature transcripts ready for translation. The function of oligo-adenylation in dinoflagellate mitochondria is unknown (other than its inclusion in *cox3* splice products), but it is consistently present in mitochondrial transcripts of both dinoflagellates and apicomplexans suggesting it does not serve as a cue for mRNA degradation as for some other organelle systems [18], [29], [32]–[34]. While RNA editing is a necessary process of mRNA maturation in dinoflagellate mitochondria [35], [36], we have previously shown that *K. veneficum cox3H1-6* precursors are fully edited [17]. Instances of minor incomplete editing were observed in some of these *cox3H1-6* transcripts, however this was also seen for *cob* transcripts (which are not trans-spliced), and appears to be a general feature of RNA editing [17]. It is, therefore, unclear how the abundant presence of these immature transcripts is managed. One possibility is that the precursor transcripts might be translated into partial Cox3 proteins that either function autonomously or are subsequently joined as proteins. Dinoflagellate mitochondria are known to be able to use alternative initiator and terminator translation signals [18], [19], so the lack of conventional open reading frames in the *cox3H1-6* and *cox3H7* transcripts might not be a barrier to translation (we have attempted to characterize Cox3 protein species by mass spectrometry but without success). However, if such novel routes to Cox3 function were viable, independent evolution of the trans-splicing process would be unnecessary. Thus we find such a scenario of partial Cox3 synthesis unlikely, although how it is avoided remains a conundrum.

**Figure 3:**
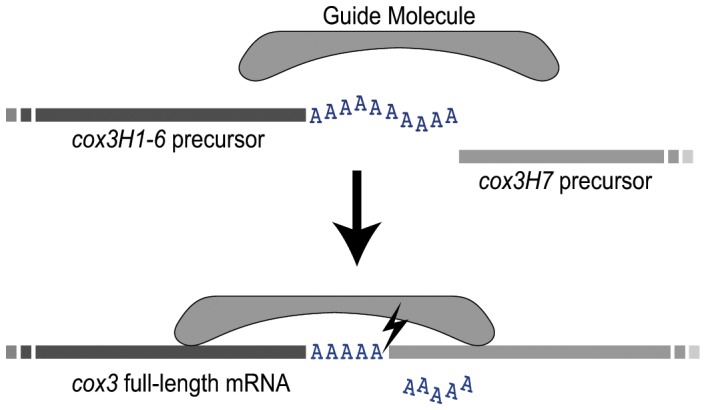
Model of *cox3* trans-splicing mechanism. Putative splicing mechanism employing a guide molecule that unites the two *cox3* precursor transcripts, and determines the length of the final splice product by inclusion of the necessary number of A nucleotides from the oligoadenylated tail of *cox3H1-6*.

The presence of a conserved splice site across diverse dinoflagellates suggests that this trait was acquired relatively early in dinoflagellate radiation, although after divergence of deep-branching taxa such as *Hematodinium* sp. and *Oxyrrhis* which lack *cox3* splicing [23], [24]. Further, from these data we can draw some conclusions about the mechanism of splicing. The lack of any flanking non-coding sequence in the *cox3* transcript precursors (other than the oligoadenosine tails) argues against flanking split group I/II introns mediating the splicing events, as occurs in other organelle trans-splicing systems [1]. There is also no evidence of likely RNA helix formation between the *cox3H1-6* 3′ end, and the *cox3H7* 5′ end, that could potentially mediate bulge-helix-bulge splicing as seen in some archaeal tRNAs [16]. This absence of any putative self-splicing components suggests that splicing is directed by some additional guide molecule or complex. Such a guide must: 1) identify the two component molecules (*cox3H1-6* and *cox3H7*); 2) define the correct length of final spliced product, allowing sufficient A nucleotides from the oligoadenylated tail to close any gap; and 3) direct the splicing reaction onto the 5′ end of *cox3H7*. Such a guide could consist of a protein (or proteins), or could be a further RNA molecule similar to RNA guides employed in editing of trypanosomatid mitochondria RNAs [37]. Extensive searching for evidence of any putative RNAs with limited complementarity to both *cox3* precursors has failed to detect any candidates. A lack of conservation seen across taxa of either the position of oligoadenylation of *cox3H1-6,* or the sequence identity of the two ends to be joined, suggests that the guide molecule is tolerant of change in this region, and might interact with sequence regions more distal to the splice site (Fig. 3). The only conserved nucleotide within the immediate splicing region is a uracil found at the 5′ splice site of *cox3H7* in all four taxa surveyed, and this nucleotide may reflect a conserved feature of the splicing reaction.

A consequence of the trans-splicing mechanism in dinoflagellate *cox3*, and the inclusion of part of the *cox3H1-6* oligoadenosine tail in the spliced product, is that a variable number of A nucleotides occur at the join region. This results in one or more lysines (codon: AAA) encoded in the complete transcript (Fig. 1C). In a poly-topic membrane protein inclusion of charged residues might be expected to cause problems for membrane topology, with potential implications for protein function. However, the location of the splice site in *cox3* is between the coding regions of two membrane helices, and presumably these charged residues (and variability in protein sequence) are tolerated at this site.

Overall, these new insights into trans-splicing of dinoflagellate mitochondrial *cox3* show that it is an unusual process on multiple scores. Unlike discontinuous group I/II intron mediated trans-splicing, there is no evidence for the precursor transcripts directly contributing to the process of splicing. Thus evolution of this trans-splicing process is more likely to have developed by the introduction of a splicing capability into these mitochondria, rather than gradual corruption of an existing splicing function such as organelle intron removal. Deep-branching dinoflagellates (e.g. *Oxyrrhis* and *Hematodinium* sp.) lack trans-splicing, although they share the same very reduced set of mitochondrial genes, so there is no evidence of existing splicing capacity in mitochondria early in this lineage [23], [24]. Also unusual is that the splicing process in dinoflagellate mitochondria is imperfect. It does not always produce a seamless join between two complete gene exons, but leaves a footprint of multiple A nucleotides that has varied in length during divergence of different dinoflagellate taxa. While this is apparently tolerated in at least one position in the Cox3 gene, presumable this would not be viable in many other locations within the three proteins encoded in dinoflagellate mitochondria. Thus development of further trans-splicing events in this system might be constrained by the imperfect nature of this process.

Only one other known system displays a comparably unusual form of RNA trans-splicing - the mitochondria of diplonemid protists that belong to the supergroup Euglenozoa [38], [39]. Here, fragmented genes (up to nine pieces in the case of *cox1*) are transcribed as separate RNAs, trimmed down to only the coding sequences, and spliced together to form complete coding transcripts. A lack of flanking non-coding RNA suggests that splicing also relies on guide molecules, although in diplonemids these too are uncharacterized. Further, at one splice junction in *cox1* a non-coded run of six uracils occurs in the mature transcript, although in this case RNA insertional editing is thought to be the mechanism, as occurs in trypanosomatid relatives of diplonemids [37], [40]. While superficially similar to the case of dinoflagellate trans-splicing, the mechanism of diplonemid trans-splicing is likely to be different to dinoflagellates, and these two groups are very distantly related to one another [41]. It is interesting to note, however, that both mitochondrial trans-splicing processes have developed in lineages that undergo trans-splicing of SLs onto their nucleus-encoded mRNAs, and also possess mitochondrial RNA editing machineries that are both presumed to entail RNA cleavage and re-ligation [18], [42]. This raises the question of whether these novel forms of RNA trans-splicing might have developed under the influence of any of this existing machinery. In dinoflagellates, SL trans-splicing involves a SL transcript containing an exon/intron GU boundary, and a corresponding AG intron/exon boundary in the nascent protein mRNA. The splicing reaction is presumed to utilize the nuclear splicesomal complex [11]. The *cox3H1-6* and *cox3H7* transcripts lack flanking intron sequences, suggesting it is unlikely to be a substrate for this complex (Fig. 1). Moreover, of 72 known genes whose products comprise the spliceosome, 66 were recently identified from the *Symbiodinium* transcriptome [40]. Importing such a complex into organelles is unprecedented, and would represent a considerable evolutionary challenge. The biochemistry of RNA editing in dinoflagellate mitochondria is currently entirely unknown, so it is difficult to speculate on whether this process could have serendipitously contributed to the novel trans-splicing process found in *cox3*.

## Supporting Information

Data S1 **cRT-PCR amplicon nucleotide sequences.** Primer binding locations are underlined. Oligoadenylated tails are shown in blue. Dashes indicates gaps between outwards-facing primer pairs (unsequenced regions of the transcripts). The 15 base 5′ tag present on two of the six *K. veneficum cox3H7* amplicons is italicised.
(RTF)
