# Supplementary material for: A Widespread and Unusual RNA Trans-Splicing Type in Dinoflagellate Mitochondria
Source: PLoS One. 2013 Feb 20;8(2):e56777. doi: 10.1371/journal.pone.0056777 (PMC3577742; doi:10.1371/journal.pone.0056777)
Supplement: Data S1 — cRT-PCR amplicon nucleotide sequences. Primer binding locations are underlined. Oligoadenylated tails are shown in blue. Dashes indicates gaps between outwards-facing primer pairs (unsequenced regions of the transcripts). The 15 base 5′ tag present on two of the six K. veneficum cox3H7 amplicons is italicised. (RTF) [file pone.0056777.s001.rtf]

Supplemental data, cRT-PCR amplicon nucleotide sequences: Primer binding locations are underlined. Oligoadenylated tails are shown in blue. Dashes indicates gaps between outwards-facing primer pairs (unsequenced regions of the transcripts). The 15 base 5' tag present on two of the six K. veneficum cox3H7 amplicons is italicised. 


K. veneficum cox3H1-6

>K. veneficum cox3H1-6 cRT-PCR amplicon 1
GAATTCTATAACTTTGGGAATCAACTGCTATATTTTGGTTTTTCTAATAGTATTCATCAGGAAGTTGCATCAGGTCCATTTTGTCT----------TTTCTTTCATCTTGTCGTTGGTCTTTTTCTTCTAAGTCTTTTTTTTTGGGGTTGTTGTTTTCCTACAAAAAAAAAAAAAAAA

>K. veneficum cox3H1-6 cRT-PCR amplicon 2
GAATTCTATAACTTTGGGAATCAACTGCTATATTTTGGTTTTTCTAATAGTATTCATCAGGAAGTTGCATCAGGTCCATTTTGTCT----------TTTCTTTCATCTTGTCGTTGGTCTTTTTCTTCTAAGTCTTTTTTTTTGGGGTTGTTGTTTTCCTACAAAAAAAAAAAAAAAAA

>K. veneficum cox3H1-6 cRT-PCR amplicon 3
GAATTCTATAACTTTGGGAATCAACTGCTATATTTTGGTTTTTCTAATAGTATTCATCAGGAAGTTGCATCAGGTCCATTTTGTCT----------TTTCTTTCATCTTGTCGTTGGTCTTTTTCTTCTAAGTCTTTTTTTTTGGGGTTGTTGTTTTCCTACAAAAAAAAAAAAAAAAAA


K. veneficum cox3H7

>K. veneficum cox3H7 cRT-PCR amplicon 1
TTGTTTGGTTTTTAAATTTAAGAGTT----------AATCTTATGGTTATTTATCTTTCTATTCCTTTACTCGTTATAAAAAAAAAAAAA

>K. veneficum cox3H7 cRT-PCR amplicon 2
TTGTTTGGTTTTTAAATTTAAGAGTT----------AATCTTATGGTTATTTATCTTTCTATTCCTTTACTCGTTATAAAAAAAAAAAAAA

>K. veneficum cox3H7 cRT-PCR amplicon 3
TTGTTTGGTTTTTAAATTTAAGAGTT----------AATCTTATGGTTATTTATCTTTCTATTCCTTTACTCGTTATAAAAAAAAAAAAAA

>K. veneficum cox3H7 cRT-PCR amplicon 4
TTGTTTGGTTTTTAAATTTAAGAGTT----------AATCTTATGGTTATTTATCTTTCTATTCCTTTACTCGTTATAAAAAAAAAAAAAAA

>K. veneficum cox3H7 cRT-PCR amplicon 5
TTCCAAGAAAAGCCTTTGTTTGGTTTTTAAATTTAAGAGTT----------AATCTTATGGTTATTTATCTTTCTATTCCTTTACTCGTTATAAAAAAAAAAAAAAAA

>K. veneficum cox3H7 cRT-PCR amplicon 6
TTCCAAGAAAAGCCTTTGTTTGGTTTTTAAATTTAAGAGTT----------AATCTTATGGTTATTTATCTTTCTATTCCTTTACTCGTTATA


A. carterae cox3H1-6

>A. carterae cox3H1-6 cRT-PCR amplicon 1
TCCTGGATGCCAATTTACTTAAAAAGATTATATTATTGGCATCAGGAAGTTGGATCAGGTCAATTTTGTCTTCTAATTAATTCTCCATGGTTATATATATTTGCTCTATACCTATTATTATCTCAACTTACATTTTATTATGATAAATCAATATTAGTTTCTAGTCTTTCTAATTATATACTCTTGTATCTTACCTCATTATCATTATTAATTTATTTATGTTATTATTATATTGATTTAATTAGAGAATGTTATAAGAAATATGAAATATTATATCCTGTTTTATTCTATGTATTTTGC----------TTTCTTTCACCTTATTGTTGGTCTTCTTCTTTTAAGTCTTCTATTTTGGGGTTGTAGTTACTTATCAAACTTAAAAAAAAAAAAAAAAA

>A. carterae cox3H1-6 cRT-PCR amplicon 2
TCCTGGATGCCAATTTACTTAAAAAGATTATATTATTGGCATCAGGAAGTTGGATCAGGTCAATTTTGTCTTCTAATTAATTCTCCATGGTTATATATATTTGCTCTATACCTATTATTATCTCAACTTACATTTTATTATGATAAATCAATATTAGTTTCTAGTCTTTCTAATTATATACTCTTGTATCTTACCTCATTATCATTATTAATTTATTTATGTTATTATTATATTGATTTAATTAGAGAATGTTATAAGAAATATGAAATATTATATCCTGTTTTATTCTATGTATTTTGC----------TTTCTTTCACCTTATTGTTGGTCTTCTTCTTTTAAGTCTTCTATTTTGGGGTTGTAGTTACTTATCAAACTT

>A. carterae cox3H1-6 cRT-PCR amplicon 3
TCCTGGATGCCAATTTACTTAAAAAGATTATATTATTGGCATCAGGAAGTTGGATCAGGTTAATTTTGTCTTCTAATTAATTCTCCATGGTTATATATATTTACTCTATACCTATTATTATCTCAACTTACATTTTATTATGATAAATCAATATTAGTTTCTAGTCTTTCTAATTATATACTCTTGTATCTTACCTCATTATCATTATTAATTTATTTATGTTATTATTATATTGATTTAATTAGAGAATGTTATAAGAAATATGAAATATTATATCCTGTTTTATTCTATGTATTTTGC----------TTTCTTTCACCTTATTGTTGGTCTTCTTCTTTTAAGTCTTCTATTTTGGGGCTGTAATTACTTATCAAACTAAAAAA


A. carterae cox3H7

>A. carterae cox3H7 cRT-PCR amplicon 1
TAGATAAATATGTATGTTTTAGAAGTTCAGAAGTACATTTATTTTTTGCTTG----------TTTATTGGCATTTTGTTGAGGTCTTATGGTTATTCATTTTTCTAGGAATTTATTTAAATT


A. catenella cox3H1-6

>A. catenella cox3H1-6 cRT-PCR amplicon 1
TTTTTTCTTTCATTCTTTTCATCACACTCGAGTTTTATTGTCTTTATCTCTGCCTGGAGACTTGTCTTTTGGAATTCTATTCATCTGGAAGTTGCATCAGATT----------TTTCTTTCATCTTGTCGTTGGTCTTTTTCTTCTAGGTCTTTTCTTTTGGGGTTGTAGTTTTCCATTAAAAAAAAAAAAAAAA


A. catenella cox3H7

>A. catenella cox3H7 cRT-PCR amplicon 1
TTCTTTCATTAGTAACCTTAAGAGTTTCAGAAGTTCATTTATTTTATAATC----------AATTTCTATTGGCATTTTCTTGAAATCTTATGGATATTTATCTTTCTAGTCTTTTACAAATCTTAAAAAAAAAAAAAAAAA

>A. catenella cox3H7 cRT-PCR amplicon 2
TTCTTTCATTAGTAACCTTAAGAGTTTCAGAAGTTCATTTATTTTATAATC----------AATTTCTATTGGCATTTTCTTGAAATCTTATGGATATTTATCTTTCTAGTCTTTTACAAATCTTAAAAAAAAAAAAAAAAAA

>A. catenella cox3H7 cRT-PCR amplicon 3
TTCTTTCATTAGTAACCTTAAGAGTTTCAGAAGTTCATTTATTTTATAATC----------AATTTCTATTGGCATTTTCTTGAAATCTTATGGATATTTATCTTTCTAGTCTTTTACAAATCTTAAAAAAAAAAAAAAAA

>A. catenella cox3H7 cRT-PCR amplicon 4
TTCTTTCATTAGTAACCTTAAGAGTTTCAGAAGTTCATTTATTTTATAATC----------AATTTCTATTGGCATTTTCTTGAAATCTTATGGATATTTATCTTTCTAGTCTTTTACAAATCTTAAAAAAAAAAAAA

>A. catenella cox3H7 cRT-PCR amplicon 5
TTCTTTCATTAGTAACCTTAAGAGTTTCAGAAGTTCATTTATTTTATAATC----------AATCTTATGGTTATTTATCTTTCTAGTCTTTTACAAATCTTAAAAAAAAAAAAAA

>A. catenella cox3H7 cRT-PCR amplicon 6
TTCTTTCATTAGTAACCTTAAGAGTTTCAGAAGTTCATTTATTTTATAATC-----------AATCTTATGGTTATTTATCTTTCTAGTCTTTTACAAATCTTAAAAAAAAAAAAAAAA

>A. catenella cox3H7 cRT-PCR amplicon 7
TTCTTTCATTAGTAACCTTAAGAACTTCAGAAGTTCATTTATTTTATAATC----------AATTTCTATTGGCATTTTCTTGAAATCTTATGGATATTTATCTTTCTAGTCTTTTACAAATCTTA

>A. catenella cox3H7 cRT-PCR amplicon 8
TTCTTTCATTAGTAACCTTAAGAGCTTCAGAAGTTCATTTATTTTATAATC----------AATTTCTATTGGCATTTTCTTGAAATCTTATGGTTATTTATCTTTCTAGTCTTTTACAAATCTTA


A. catenella sp. cox3 full
>A. catenella cox3full cRT-PCR amplicon 1
TTTTTTCTTTCATTCTTTTCATCACACTCGAGTTTTATTGTCTTTATCTCTGCCTGGAGACTTGTCTTTTGGAATTCTATTCATCTGGAAGTTGCATCAGATT----------TTTCTTTCATCTTGTCGTTGGTCTTTTTCTTCTAGGTCTTTTCTTTTGGGGTTGTAGTTTTCCATTAAAAATTCTTTCATTAGTAACCTTAAGAGTTTCAGAAGTTCATTTATTTTATAATTTACAACTTTTCTATTGGCATTTTCTTGAAATCTTATGGATATTTATCTTTCTAGTCTTTTACAAATCTTAAAAAAAAAAAAA


Symbiodinium sp. cox3H1-6

>Symbiodinium sp. cox3H1-6 cRT-PCR amplicon 1
TTTTTTCGTTGGTATTCTTTCACCTTGGTATTTTGGAAATCTAAACATTTAGAAGTTGCATCAGGTGCATTTTGTCT----------TTTCTTTCATCTTGTCGTTGGTATTTTACTTCTTTTTCTTATCTTCTTTCTGGGTTCTTTTAAAAAAAAAAAAAAAA


Symbiodinium sp. cox3H7

>Symbiodinium sp. cox3H7 cRT-PCR amplicon 1
TGTCACACTTTATAAACTTTAGAATATCAGAAGTTCATTTATTTTATAATC----------AATTTCTATTGGCATTTTCTTGAAATCTTATGGTTATTTATCTTTCTAGTCTTTTATCTTTAAAAAAAAAAAAAAA

>Symbiodinium sp. cox3H7 cRT-PCR amplicon 2
TGTCACACTTTATAAACTTTAGAATATCAGAAGTTCATTTATTTTATAATC----------AATTTCTATTGGCATTTTCTTGAAATCTTATGGTTATTTATCTTTCTAGTCTTTTATCTTCAAAAAAAAAAAAAAAA

>Symbiodinium sp. cox3H7 cRT-PCR amplicon 3
TGTCACACTTTATAAACTTTAGAATATCAGAAGTTCATTTATTTTATAATC----------AATTTCTATTGGCATTTTCTTGAAATCTTATGGTTATTTATCTTTCTAGTCTTTTATCTTTAAAAAAAAAAAAAAAAAAA

>Symbiodinium sp. cox3H7 cRT-PCR amplicon 4
TATAAACTTTAGAATATCAGAAGTTCATTTATTTTATAATC----------AATTTCTATTGGCATTTTCTTGAAATCTTATGGTTATTTATCTTTCTAGTCTTTTATCTTTAAAAAAAAAAAAAAA

>Symbiodinium sp. cox3H7 cRT-PCR amplicon 5
TGTCACACTTTATAAACTTTAGAATATCAGAAGTTCATTTATTTTATAATC----------AATTTCTATTGGCATTTTCTTGAAATCTTATGGTTATTTATCTTTCTAGTCTTTTATCTTTAAAAAAAAAAAAAAA

>Symbiodinium sp. cox3H7 cRT-PCR amplicon 6
TGTCACACTTTATAAACTTTAGAATATCAGAAGTTCATTTATTTTATAATC----------AATTTCTATTGGCATTTTCTTGAAATCTTATGGTTATTTATCTTTCTAGTCTTTTATCTTTAAAAAAAAAAAAAAAA

>Symbiodinium sp. cox3H7 cRT-PCR amplicon 7
TGTCACACTTTATAAACTTTAGAATATCAGAAGTTCATTTATTTTATAATC----------AATTTCTATTGGCATTTTCTTGAAATCTTATGGTTATTTATCTTTCTAGTCTTTTATCTTTAAAAAAAAAAAAAAAA


Symbiodinium sp. cox3 full
>Symbiodinium sp. cox3full cRT-PCR amplicon 1
AATTTCTATTGGCATTTTCTTGAAATCTTATGGTTATTTATCTTTCTAGTCTTTTATCTTTAAAAAAAAAAAATTTTTTCGTTGGTATTCTTTCACCTTGGTATTTTGGAAATCTAAACATTTAGAAGTTGCATCAGGTCCATTTTGTCTTCTTATTAATAGTCCTTGGTTATTAATCTTTGCTCTTATTATAGTTCTATTTATCTTTACAGGATTTAATATTTATTGTTGGGCTGGAATTCACTTCTCATGGAGTTATTCTCTTATACTTTGTCATCTTTTATTTGGATTAATTTATAGTTTTAATATTTGGTCTAGAGATTTATTAAGAGAATTCACTAAAAAATATGAAATCTTATTAATGGTCTTTTTTCTTCTTTTTGGGGGTTTTCTAGCTTCTGAAGCTCTATTATTTATATCCTTCTTTTGGACATCTTTTCATTTATTATCTTCTCCAACTTTAGGGATGTGGCCTGGAGAAGCTTTCTATCTTCCAGATCCTTGTGAATTAACTTTTGCTAATACACTTCTTTTATCTAATGCTGCTGTATCTTTAGGAGGTGCTTTTGTTAGTTTAGAAATTTCATCACAATATATTATTTTCTTCGCTTTGTGGTCATTCTGTTTATCTTCTCTTTTTATTAGTTTGCAGATTAAAGAATTTCGTATTCTCGCATATTCAATTAATGATTCACTTTATAGTTGTCTTTTCTTTTTTCTTACAGCATTACATTTCTTTCATCTAATTCTTGGTATTTTACTTCTTTTTCTTATCTTCTTTCTGGGTTCTTTTAAAAAAAAAATGTCACACTTTATAAACTTTAGAATATCAGAAGTTCATTTATTTTATAATC
